# 3D printing for congenital heart disease: a single site’s initial three-yearexperience

**DOI:** 10.1186/s41205-018-0033-8

**Published:** 2018-11-08

**Authors:** Justin Ryan, Jonathan Plasencia, Randy Richardson, Daniel Velez, John J. Nigro, Stephen Pophal, David Frakes

**Affiliations:** 1 Rady Children’s Hospital–San Diego, San Diego, CA USA; 2 Phoenix Children’s Hospital, Phoenix, AZ USA; 3 Arizona State University, Tempe, AZ USA; 4 St. Joseph’s Hospital and Medical Center, Phoenix, AZ USA

**Keywords:** Congenital heart disease, 3D printing, Retrospective chart review, Patient outcomes

## Abstract

**Background:**

3D printing is an ideal manufacturing process for creating patient-matched models (anatomical models) for surgical and interventional planning. Cardiac anatomical models have been described in numerous case studies and journal publications. However, few studies attempt to describe wider impact of the novel planning augmentation tool. The work here presents the evolution of an institution’s first 3 full years of 3D prints following consistent integration of the technology into clinical workflow (2012–2014) - a center which produced 79 models for surgical planning (within that time frame). Patient outcomes and technology acceptance following implementation of 3D printing were reviewed.

**Methods:**

A retrospective analysis was designed to investigate the anatomical model’s impact on time-based surgical metrics. A contemporaneous cohort of standard-of-care pre-procedural planning (no anatomical models) was identified for comparative analysis. A post-surgery technology acceptance assessment was also employed in a smaller subset to measure perceived efficacy of the anatomical models. The data was examined.

**Results:**

Within the timeframe of the study, 928 primary-case cardiothoracic surgeries (encompassing both CHD and non-CHD surgeries) took place at the practicing pediatric hospital. One hundred sixty four anatomical models had been generated for various purposes. An inclusion criterion based on lesion type limited those with anatomic models to 33; there were 113 cases matching the same criterion that received no anatomical model. Time-based metrics such as case length-of-time showed a mean reduction in overall time for anatomical models. These reductions were not statistically significant. The technology acceptance survey did demonstrate strong perceived efficacy. Anecdotal vignettes further support the technology acceptance.

**Discussion & conclusion:**

The anatomical models demonstrate trends for reduced operating room and case length of time when compared with similar surgeries in the same time-period; in turn, these reductions could have significant impact on patient outcomes and operating room economics. While analysis did not yield robust statistical powering, strong Cohen’s d values suggest poor powering may be more related to sample size than non-ideal outcomes. The utility of planning with an anatomical model is further supported by the technology acceptance study which demonstrated that surgeons perceive the anatomical models to be an effective tool in surgical planning for a complex CHD repair. A prospective multi-center trial is currently in progress to further validate or reject these findings.

## Background

Congenital heart disease (CHD) is a significant morphological deviation of cardiac anatomy present at birth, resulting in hemodynamic and functional anomalies, often necessitating early interventional and/or surgical palliation or repair. Patients with CHD lesions represent a significant part of the medical population as the lesions are present in approximately 8 out of 1000 births in the United States [1–3] and represent the leading cause of mortality from congenital defects [4].

Imaging modalities used for diagnosis and treatment planning are computed tomography (CT), magnetic resonance (MR) imaging, and echocardiography (echo). Medical image post-processing and volumetric rendering techniques provide a wealth of information pre- and peri-procedural planning; however, the images remain separated from the physical domain in which the surgeons actively work. Three-dimensional (3D) printing enables patient-matched (also known as patient-specific) anatomical models, giving clinicians an opportunity to view anatomy and lesions specific to a patient at a given point in time.

3D printing is an ideal manufacturing process enabling reproduction of patient-matched morphology in a physical manner due to its additive methods. 3D printing of cardiac anatomy (hereinafter referred to as “anatomical models”) for surgical planning was described in journals as early as 2000 [5]. The medical applications have proliferated in the last 10 years; a recent white paper by SME (formerly Society of Manufacturing Engineering) states that in 2016 approximately 99 institutions produced 3D models on site (point-of-care manufacturing) [6]. The explosion and adoption of this technology has yielded a wealth of clinical cases wherein care was augmented by 3D printed cardiac models [5, 7–37]. In the domain of CHD lesions, a recent publication by Yoo et al. effectively details the methods of creating CHD anatomical models [38]. It is worth noting that only a select number of studies statistically describe impact of the still-novel technology [36, 38, 39]. Adoption of 3D printed models for morphologically-complex CHD anatomy is on the rise; clinical trials will further validate its efficacy.

The work here presents the evolution of an institution’s first 3 full years of 3D prints following consistent integration of the technology into clinical workflow (2012–2014) – a center which has produced over 500 to date. A retrospective analysis was performed over time-based metrics relating to patient outcomes. Contemporaneous cohorts of standard-of-care (SoC) pre-procedural planning and 3D printing (3DP) pre-procedural planning were collected and compared. The data was statistically examined; however, statistical powering was not a central focus. The aim of this pilot study was to 1) review the impact of 3D printing within a single healthcare institution, 2) understand what metrics may serve as ideal primary endpoints for subsequent studies, and 3) inform the creation of clinical trial with 3D printing as the experimental arm. The results and conclusion of this study assisted in the formation of a prospective multi-center study investigating the efficacy of 3D printing in complex congenital heart disease.

### Technology acceptance modeling

In order to measure true potential adoption of a technology, the ease-of-use of the innovation must match its utility (i.e., the ability to help the surgical or medical care unit). Accordingly, a technology acceptance model (TAM) as described by Davis et al. is one of the established methods of examining the intent to adopt and use a new technology [40]. The novelty still surrounding 3D printing in medicine yields great opportunities for utilizing TAM to describe potential intent-to-use [41, 42].

TAM has been utilized in many fields including information technology, workforce management, and medicine [43–45]. An 88-study meta-analysis conclude that the TAM is a predictive model of behavioral intention. The model was found to be considerably more effective in describing intention when the respondents were a professional cohort – such as cardiothoracic surgeons [44]. A surgical team cohort responding to TAM surveys should yield meaningful results on the behavioral intention to use 3D printed anatomical models.

Without the perception of efficacy, defined by behavioral intention, the physical modeling of a patient’s morphology would fail to be adopted into standard clinical practice. As an adjunct to the patient outcomes data analysis, a TAM analysis was performed with participating cardiothoracic surgeons. A post-surgery assessment was implemented in the standard clinical care process, where perceived usefulness was explored.

## Methods

Phoenix Children’s Hospital’s Institutional Review Board approved the following retrospective study for patients between September 1, 2012 and December 31, 2014.

Through the course of clinical care, cardiothoracic surgeons and cardiologists at the participating pediatric hospital, identified 79 cases as candidates to receive a 3D model for surgical planning. The selection criteria were surgeons’ request (largely based on perceived complexity of lesion) and available image data. As this was not a prospective trial, specific inclusion/exclusion criteria were not utilized to determine which patients received anatomical models.

### Image acquisition

Per standard-of-care, patients received a contrast-enhanced CT or MR scan. Spatial resolution of the image datasets varied from patient-to-patient. CT slice thickness (related to resolution in z-direction) ranged from 0.325 mm to 0.9 mm; pixel spacing (resolution in x- and y-direction) ranged from 0.325 mm to 0.625 mm. The MRI datasets lacked the spatial resolution found in the CT datasets with voxel dimensions as large as 2.5 mm. 3D echo was not investigated in the early years of the lab; no patients in the retrospective analysis had anatomical models generated from echo.

### Segmentation and reconstruction

The image datasets were imported into Mimics Innovation Suite (Materialise, Lueven, Belgium), a medical image processing software suite. The software facilitated image segmentation, the process of partitioning regions of an image into discrete sections. The segmentation was largely achieved with intensity value thresholding followed with semi-automated and manual segmentation. For common cardiac anatomy, the following blood volume subsets were segmented: left atrium with pulmonary veins, right atrium with vena cava, left ventricle, right ventricle, pulmonary arteries, aorta, and coronary arteries. Additional segments included the CHD lesions (e.g., patent ductus arteriosus, collateral vessels, fistulas, etc.). The segmented masks were reconstructed into 3D surface mesh models.

The computational cardiac anatomies were then imported into 3D engineering software suites, Geomagic (3DSystems, Rock Hill, SC, USA) and 3-matic (Materialise, Lueven, Belgium), for additional post-processing. Reconstruction artifacts such as “stair-stepping” was minimized through noise removal and mesh reconstruction. As greater smoothing is performed, model detail may be lost. Qualitative accuracy was maintained by comparing the modified geometry against the source images; contours of the geometry were projected on the orthogonal slices. A multidisciplinary team qualitatively affirmed accuracy prior to proceeding by reviewing the reconstruction in conjunction with the underlying image dataset(s).

The models advanced to a coloring step to best utilize the human visual system. A contemporary study in the medical educational domain assisted in the definition of a coloring scheme [39]. Cardiac components relied on a red-blue coloring scheme based on a normal morphology; morphologic right-sided structures received blue hues, while morphologic left-sided structures received a red hue. The morphologic color scheme was maintained even in cases of transposition, dextro-position, heterotaxy, etc. As part of standard operating protocols, all models were labeled with a unique identifier disassociated to the patient’s medical record number.

### 3D printing

The color-coded computational models were produced in-house with a gypsum-based, binder-jetting 3D printer: zPrinter 650 (3D Systems, Rock Hill, SC, USA). This 3D printing technology uses a full cyan-magenta-yellow-key-ink (CMYK-ink), cyanoacrylate infiltration system. The printer deposits a 0.1 mm thick, flat layer of gypsum powder on a build platform resulting in a hard resin model. Print heads jet a binding agent and colorant onto the gypsum layer. Models are manually removed from support powder and coated with cyanoacrylate for added durability.

**Fig. 1:**
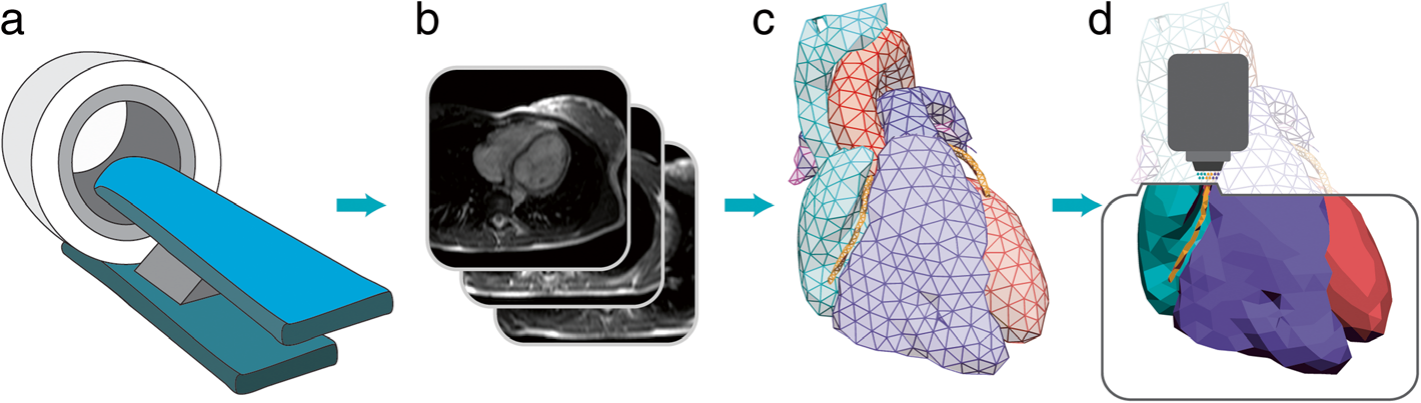
An overview of the anatomical model creation process: **a** the patient receives a CT or MRI scan producing **b** slice images; **c** the images are reconstructed into a 3D computational model; and **d** the computational model is printed with a 3d printer. Please see the 2016 publication by Yoo et al. for a thorough review of the anatomical modeling process

Each final 3D printed anatomical model used for surgical planning went through an iterative design process where a radiologist or cardiologist qualitatively assessed color-coding and anatomical accuracy at each modeling stage. When errors were encountered, the models were adjusted accordingly and rechecked. For an in-depth review of the anatomical model methods for congenital heart disease, see the 2016 publication by Yoo et al. [38] A simplified illustration of the modeling process can be seen in Fig. 1.

### Data analysis

The retrospective chart review was performed over all patients within the described time frame that had cardiothoracic surgery. Statistical software (JMP, SAS Institute, Cary, NC) facilitated analysis. Due to the literature-based correlation of surgical length of time to morbidity and mortality, the effects of anatomical models on patient outcomes was analyzed via a one-way analysis of variance (ANOVA) with effect size further analyzed with Cohen’s *d*. Response variables included 1) operating room length of time (in minutes), defined as the time differential from when the patient is wheeled into the operating room to the time he or she is wheeled out and 2) case length of time (minutes), the duration of the surgery. Direct morbidity and mortality was analyzed via contingency tables (with Fisher’s exact test). Response variables included 1) 30-day readmission (yes/no), the binary response whether the patient had to be readmitted to the hospital within 30 days of hospital discharge, and 2) 30 day mortality (yes/no), the binary response whether the patient died post-surgery within 30 days of hospital discharge. The data followed the Society of Thoracic Surgery nomenclature for lesions and patient outcome metrics.

Following a surgery, the cardiothoracic surgeon completed a TAM-questionnaire. Four questions, seen in Table 5, were established using a basic TAM model [40, 41]. The data was also analyzed with statistical software.

## Results

Within the timeframe of the study, 928 primary-case cardiothoracic surgeries (encompassing both CHD and non-CHD surgeries) took place at the practicing pediatric hospital. In that time-frame, 164 anatomical models had been generated for various purposes: education, family consultation, catheter-based intervention, and surgical planning; 79 models were specifically used for surgical planning of CHD patients. As these 79 spanned many different disease lesions with drastically different inherent complexities, a further-restricting inclusion criteria was established keeping patients with the following lesions: 1) pulmonary atresia (ventricular septal defect variant), 2) Tetralogy of Fallot (pulmonary atresia and absent pulmonary valve variants), 3) double outlet right ventricle (transposition of the great arteries variant), 4) truncus arteriosus, 5) vascular rings, and 6) single ventricle. The inclusion criteria limited those with anatomic models to 33; standard-of-care (no anatomical models) cases for the same time frame and inclusion criteria amounted to 113 (Tables 1, 2 and 3).

**Table 1:** ANOVA table illustrating the effect of anatomical model-based planning on case length of time. Green cells illustrate the lower, preferred mean time for surgeries planned with an anatomical model. Anatomical models are abbreviated as 3DP. Abbreviations: D.F. is degrees of freedom, Adj. S.S. is adjusted sum of squares, Adj. M.S. is adjusted mean squares, St.Dev is standard deviation, and C.I. is confidence interval

Case length of time (anatomical model vs Traditional Planning): all included patients					
Source	D.F.	Adj. S.S.	Adj. M.S.	F-Value	*P*-Value
3DP	1	1916	1916	0.18	0.674
Error	144	1,557,292	10,815		
Total	145	1,559,208			
Planning	N	Mean (minutes)	St.Dev	95 C.I.	
SoC	113	229.33	101.81	(209.99, 248.66)	
3DP	33	220.7	111.3	(184.9, 256.4)	

Case length of time’s Cohen’s *d* effect size was small (0.081) suggesting no practical difference between SoC and 3DP case length of time for all patient cases

**Table 2:** Contingency tables illustrating the effect of anatomical model-based planning on 30-day readmission and 30-day mortality. Fisher’s exact test was used to determine probability for the rejection of the stated null hypothesis

30-day Readmission (anatomical model vs Traditional Planning): all included patients				
Count Total%	No 30-day Readm.	30-day Readm.	Total	Fisher’s Exact Test
SoC	31 22.30%	78 56.12%	109 78.42%	Null Hypothesis: • Probability of readmission is greater for surgeries planned with an anatomical model • *P*-value = 0.1609
3DP	12 8.63%	18 12.95%	30 21.58%	
Total	43 30.94%	96 69.06%	139 100.0%

**Table 3:** Contingency tables illustrating the effect of anatomical model-based planning on 30-day readmission and 30-day mortality. Fisher’s exact test was used to determine probability for the rejection of the stated null hypothesis

30 day Mortality (anatomical model vs Traditional Planning): all included patients				
Count Total%	No 30-day Mort.	30-day Mort.	Total	Fisher’s Exact Test
SoC	111 76.03%	2 1.37%	113 77.40%	Null Hypothesis: • Probability of 30-day mortality is greater for surgeries planned with an anatomical model • P-value = 0.5978
3DP	33 22.60%	0 0.00%	33 22.60%	
Total	144 96.63%	2 1.37%	146 100.0%	

It is worth noting that Table 2 illustrates a 71.6% readmission rate for patient in SoC (for this specific patient cohort) while readmission rates for patients with anatomic models was only 60%; statistical significance with the conventional *p*-value of 0.05 was not achieved.

While ANOVA and Fisher’s exact test failed to illustrate the anatomical models’ effect with a p-value less than 0.05, every response variable trended toward more favorable outcomes. Even with the restrictive nature of the exclusion criteria, surgeries were further blocked to better estimate anatomical models’ effect for specific diagnoses. For example, double outlet right ventricle (DORV) presenting with the transposition of the great arteries (TGA) variation cases and well as truncus arteriosus cases were analyzed independent of other cases with regards surgical time response variables (Tables 4 and 5).

**Table 4:** ANOVA tables for the effect of anatomical model in planning for DORV-TGA cases. Response variable is case length of time

Case length of time (Anatomical Model vs Traditional Planning): DORV (TGA-type)					
Source	D.F.	Adj. S.S.	Adj. M.S.	F-Value	*P*-Value
3DP	1	26,368	26,368	1.88	0.207
Error	8	111,962	13,995		
Total	9	138,330			
Planning	N	Mean (minutes)	St.Dev	95 C.I.	
SoC	8	359.4	118.4	(262.9, 455.8)	
3DP	2	231.0	117.4	(38.1, 423.9)	

Case length of time’s Cohen’s *d* effect size was large (1.098) suggesting a practical difference between SoC and 3DP case length of time for DORV patient cases. This large effect size further suggests the study p-value was likely poor because the study population size not due to poor trends

**Table 5:** ANOVA tables for the effect of anatomical model in planning for truncus cases. Response variable is case length of time

Case length of time (anatomical model vs Traditional Planning): Truncus					
Source	D.F.	Adj. S.S.	Adj. M.S.	F-Value	P-Value
3DP	1	11,267	11,267	1.47	0.271
Error	6	45,959	7660		
Total	7	57,226			
Planning	N	Mean (minutes)	St.Dev	95 C.I.	
SoC	6	321.7	95.9	(234.2, 409.1)	
3DP	2	235.00	1.41	(83.57, 386.43)	

Case length of time’s Cohen’s *d* effect size was very large (1.278) suggesting a practical difference between SoC and 3DP case length of time for Truncus patient cases. Similar to the DORV patients, this very large effect size strongly suggests the poor *p*-value may be related to the study population size, not the variance or mean difference

The effect of anatomical models on DORV (TGA-type) and Truncus Arteriosus yielded mean difference greater than the mean difference across all of the cases. Even with the greater mean difference, the ANOVA analysis still failed to yield a p-value less than 0.05; an additional note is that the sample size for each analysis, especially after the additional lesion-based blocking, was considerable small in size.

### Technology acceptance results

The post-operative TAM survey was placed into the clinical care process on October 14, 2014 through the end of the year. Nineteen cases received survey responses with 4 cases planned with an anatomical model.

Favorable responses outweighed other responses in the TAM questions. The average TAM score was significantly higher in the perceive usefulness domain as evidenced by the responses to TAM question 2 as seen in Table 6. Responses to TAM questions 3 and 4 supported both perceived usefulness and perceived ease-of-use through free text responses.

**Table 6:** Technology acceptance model survey responses for the utility of anatomical models for surgical planning

Technology Acceptance Model Survey (19 responses)		
Question	%	Answer
1. Was a 3D printed model used for the preparation of or during surgery/intervention? (19 applicable cases)	21.1%	Yes
	78.9%	No
2. In your opinion, did use of the 3D printed model enhance your ability to execute a surgical repair? (4 applicable cases)	100%	Yes
	0.00%	No
3. If no 3D model was used but CT/MR was used, did you note any additional morphological defects or unexpected variations unseen in the planning process? (14 applicable cases)	21.4%	Yes
	78.6%	No
4. Please provide any additional information describing the impact of the 3D printed model during the planning or execution of this patient’s surgery?	(free text response) See section Discussion	

TAM question 1 segments the response into two cohorts based on the utilization of a 3D printed model. All responses by the surgeons revealed strong support for anatomical models which addresses the perceived usefulness as described in the TAM model. In addition, question 3 asks “If no 3D model was used but CT/MR was used, did you note any additional morphological defects or unexpected variations unseen in the planning process?” Of the available 14 responses, surgeons recognized 3 cases where additional or unexpected anatomical presentation occurred.

There existed no variance in the efficacy of anatomical models due to the lack of negative responses; no ANOVA testing was possible. The anecdotal data provided by question 4 will be discussed in the Discussion section due to potential bias created by personal opinions.

## Discussion

The advent of commercially-available 3D printing technology has enabled systematic development of anatomical models for surgical planning. The retrospective study illustrates not only the systemic integration of 3D technology into the medical environment, but it also proposes potential areas of impact within the care system. However, the work herein is not unique to the medical domain. As mentioned, there are numerous publications from case studies to case series and even a few larger studies already in print [5, 7–37]. The novelty of this study is in its earnest attempt to statistically describe the anatomical models’ impact with the intent of informing the design of a subsequent studies/trials.

The various ANOVA tables illustrated consistent reduction in operative time metrics when planned with an anatomical model. Every table illustrates that mean time for the operating room and case length were less when the case was planned with an anatomical model with highly complex disease lesions mean time greater than 90 min. As is suggested with the effect sizes (assuming the sample means and standard deviations would hold if the study population size was increased), the large *p*-values for the complex congenital cases were a likely consequence of the small study sizes for each of the disease types.

The reduction of these durations may lead to lower morbidity and mortality, especially through the reduction of duration-associated infections [46]. While patient safety and good outcomes are the primary aim for any medical and surgical procedure, it is important to note the economic impact of any new technology into the clinical/surgical workflow. A negative impact may hinder technological adoption. Costs related to morbidity post-surgery are offset by the patient/patient-family, insurance companies, and hospitals. In addition, the time allocated for an operation has an associated cost; either a direct cost per time unit or indirect cost per procedure (depending on the hospital’s business model) [47–49]. By reducing the time an operation takes, the hospital will save both money and resources that would have likely been consumed in a longer operation. The Mayo Clinic has stated in various presentations that one minute of operative time is equivalent to $80–150; several minutes saved in the operative room can translate to cost coverage of the production of an anatomic model. If the time saved is great enough, additional surgeries can take place in a single day, further adding to the economic incentive of anatomical models for complex cases. It is critical to note that while mean time reductions were observed – and in complex lesions the mean difference was quite dramatic – statistical significance was never established. An explanation of this may be due to low sample size and/or high variance (see Section Future Work for a response to this).

The results of the TAM surveys suggest that anatomical models are becoming an accepted new technology. Responses to question 2 revealed positive behavioral attitude to the technology, supporting intention to use anatomical models in standard of care. The other critical component to TAM models, perceived ease-of-use, was not measured in the study as there was not additional work needed from the medical unit. The additional work and resources to generate the models were supported through academic collaborations and philanthropic grants. Investigating ease-of-use will need to be further examined when 3D printing utilizes direct resources from an institution. To better understand the potential intention to use (related here to the usefulness as described by TAM), responses to qualitative questions 3 and 4 were analyzed. Findings from the responses suggest that anatomical models benefited the cardiothoracic surgeons in several key areas: 1) improved spatial acuity, 2) improved surgical planning, 3) addressed deficits from traditional medical imaging.

In a case featuring complex Tetralogy of Fallot, the surgeons positively responded that the anatomic model facilitated spatial acuity related to critical structures:

“[W]e needed to understand the relationship between the pulmonary arteries and the anomalous coronary arteies [sic], and needed to plan the reconstruction of the [right ventricular outflow tract]. [T]he 3D model helped understand these [relationships]”.

The planned intervention for this specific patient was a surgical palliation by placing a shunt between the aorta and pulmonary artery. The shunt’s intention is to circumvent the pulmonary obstruction; however, a common complication is the partial obstruction of coronary vessels (depending on the presence and course of those vessels). This patient featured an aberrant coronary pathway; the anatomical model afforded the surgeons the patient-matched information prior to opening the patient.

The positive response to the anatomical model was not unique. The perceived improvement in spatial acuity also facilitated improved patient-matched surgical plans:

“[The anatomical model] helped delineate all anatomic relationships and specifically the pulmonary veins which were difficult to see on echo. The model was also useful to determine where we would place the Glenn/how to perform the surgery.”

This response not only shows the improved understanding of the spatial relationships, but also specifically points to deficiencies in other imaging modalities. Specifically, the anatomical model revealed structures unseen in the echo due to the patient’s diminutive size and low pulmonary blood flow. In addition, the improved understanding between the superior vena cava and the pulmonary artery is essential for the Glenn procedure; the model anecdotally facilitated planning. These vignettes illustrate the perceived benefits of the anatomical models due to their capability of representation morphologically complex disease lesions. Similar examples of morphologically complex lesions such as double outlet right ventricle are found in contemporary literature [38].

The anatomical models measured effects on planning (the ANOVA tables) and surgeon intention (the anecdotal vignettes) illustrate the role the anatomical model had in establishing the surgeons’ spatial understanding of the patient’s anatomy by presenting an accurate, absolute-scale reference for the specific patient. In addition, the use of anatomical model potentially reduces surgical and operating room length of time for complex surgeries. Morbidity and mortality are suggested to be linked to these time metric [46], a reduction of these times due to effective planning may possible with anatomical models. While the ANOVA tables and associated TAM vignettes demonstrate the efficacy of the anatomical model, the process and final model have limitations.

### Limitations

This study is a retrospective review, not a clinical trial, as such there were limitations in the methodology for minimizing confounding factors. Patient inclusion/exclusion criteria, imaging protocol, and surgeons all provide numerous avenues for confounding data analyses and limiting bias. These limitations are well described in literature relating to studies and trials; the effect of these limitations will be described [50].

The initial barrier encountered is common to many pilot/early studies: limited sample size. Initially, the study pool started at 79 cases; however, this patient population spanned complexity of disease lesions. Comparing complex lesions to comparatively simple lesions with regards to patient outcomes would not likely yield compelling analyses or worthwhile discussion. An early decision was to limit the study population to complex lesions (*n* = 33) for later ANOVA analyses. In addition, the method of analysis compared time-based metrics between surgeries planned with anatomical model and standard-of-care procedures. The analyses were further blocked by diagnoses; however, this blocking does not account for other lesion complexity factors such as syndromes, additional congenital defects, or prior surgeries. To limit biases related to these potentially confounding factors, a study would need a much more-narrow scope for disease lesion and surgical repair or a much greater patient population to facilitate more meaningful blocking. Neither solution was pragmatic for this retrospective study; however, it may be utilized for planning a prospective clinical trial. Even with these further limitations/blocking in a prospective study design, any study on anatomical models would be challenged by remaining, uncontrolled nuisance factors.

As for the TAM study, the two participating cardiothoracic surgeons, while not principal investigators, were aware that the intent of the study: understanding the impact of anatomical models on surgical outcomes; therefore, a detection bias may have been present [50]. A detection bias can occur when the recording of an outcome is subconsciously affected by the participants’ preconceptions. Post-surgical responses are an observation of the surgical planning process after the completion of a surgery. Observations made in this manner may also yield the detection bias as the surgeon may subconsciously be looking for additional benefits of the anatomical model or disassociating adverse surgical events from the anatomical model. Evidence of this bias may be present in the 100% positive response rate to the anatomical models perceived efficacy. Without a larger sample size and stricter inclusion protocols, the potential for detection bias is difficult to control. A multi-site study with pre-, peri-, and post-surgical assessments could further limit the detection bias while also revealing more of the habits-of-mind behind surgeon interaction with an anatomical model.

### Future direction

A multi-center clinical trial was formulated based in part on the data presented in this publication. 3D Hearts Enabling a Randomized Trial (3DHEART) is a trial to gauge the efficacy of anatomical models in reducing cardiopulmonary bypass time (primary endpoint) in addition to over 20 secondary patient outcome endpoints. The data in this manuscript assisted in defining the inclusion criteria as only patients with DORV-, D-TGA-, and truncus-type anatomy.

## Conclusion

Despite these study biases and modeling limitations, the surgical anatomical model study demonstrates trends for reduced operating room and case length of time. The added benefit may be attributed to better surgeon preparedness. This preparedness may yield better patient outcomes with lower chances for morbidity and mortality. The utility of planning with an anatomical model is supported by the TAM study which demonstrated that anatomical models for surgical planning may increase surgeon familiarity of patient-specific morphology and help surgeon plan for a complex CHD repair. A multi-center clinical trial, currently in progress, could show the measured effect of the anatomical model on critical surgical factors such as 30-day outcome, case length of time, or cardiopulmonary bypass time. Illustrating reductions in morbidity and mortality in patients with CHDs would aid in the acceptance, by the greater medical community, regarding the efficacy of anatomical model as a surgical planning tool. Acceptance of the technology is already high at the participating hospital where over 500 hearts have been printed for clinical planning to date.

## Abbreviations

3D: Three-dimensional
3DP: 3D Printed
ANOVA: Analysis of variance
CHD: Congenital heart defect
CT: Computed tomography
DORV: Double outlet right ventricle
MRI: Magnetic resonance imaging
SoC: Standard of care
TAM: Technology acceptance modeling
TGA: Transposition of the great arteries
